# Acquired Gitelman Syndrome Associated with Systemic Sclerosis

**DOI:** 10.7759/cureus.3923

**Published:** 2019-01-20

**Authors:** Muhammad Masab, Abhinav Goyal, Surbhi Abrol, Janani Rangaswami

**Affiliations:** 1 Internal Medicine, West Virginia University, Morgantown, USA; 2 Internal Medicine, Einstein Medical Center, Philadelphia, USA; 3 Dentistry, Smilekraft Clinic, Dharamshala, IND

**Keywords:** acquired gitelman syndrome, systemic sclerosis, scleroderma, hypokalemic metabolic alkalosis, hypocalciuria

## Abstract

Gitelman syndrome is an inherited renal disorder characterized by hypomagnesemia, hypokalemia, hypocalciuria and metabolic alkalosis linked to the genes encoding the thiazide sensitive NaCl cotransporter (NCCT) located on the distal convoluted tubule of the kidney. It usually presents in late childhood or early adulthood with electrolyte abnormalities resembling chronic thiazide diuretic use. Acquired Gitelman syndrome is a very rare disorder mostly associated with Sjogren’s syndrome.

## Introduction

Gitelman syndrome is an autosomal recessive disorder characterized by hypokalemic, hypomagnesemic and hypocalciuric metabolic alkalosis. The clinical phenotype is a result of a mutation in the SLC12A3 gene which encodes the thiazide sensitive NaCl cotransporter (NCCT) in the distal convoluted tubule of the kidney [1]. Classic symptoms include muscle spasms, muscle cramps, weakness, fatigue and abdominal pain. Inherited Gitelman syndrome tends to present in late childhood and resembles the electrolyte disturbances seen with chronic thiazide use [2]. Acquired Gitelman syndrome is extremely rare with four reported cases to date, one post renal transplant and others secondary to Sjogren’s syndrome [2-3]. Renal tubular disorders like Gitelman syndrome and Bartter syndrome should be considered in the differential diagnosis of autoimmune diseases with electrolyte disturbances and renal dysfunction. We hereby report a case of Gitelman syndrome in a patient with systemic sclerosis.

## Case presentation

A 54-year-old woman with past medical history of systemic sclerosis presented with fatigue, muscle cramps and progressive dysphagia. She was initially diagnosed with systemic sclerosis after presenting with hypertension and scleroderma renal crisis. She denied any dryness of eyes or mouth at that time. She also had diffuse cutaneous thickening, esophageal dysmotility and Raynaud’s phenomenon. Her renal function was normal, i.e., blood urea nitrogen (BUN) of 13 mg/dL (10–20 mg/dL), serum creatinine (Cr) of 0.7 mg/dL (0.6–1 mg/dL). Anti-SSA antibody was negative at the time of initial diagnosis. After the initial episode of scleroderma renal crisis, the patient was on maintenance mycophenolate for worsening skin disease, which ultimately was tapered off due to poor response. Her hypertension was well controlled (systolic blood pressure (BP) 80–100 mmHg and diastolic BP 50–60 mmHg) on a calcium channel blocker and angiotensin converting enzyme inhibitor.

New onset hypokalemia and hypotension were noted eight years into the course of her disease, necessitating cessation of her anti-hypertensives. Her serum electrolytes and renal function tests were as follows: BUN 11 mg/dL (10–20 mg/dL), Cr 0.87 mg/dL (0.6–1 mg/dL), sodium 132 mmoL/L (136–146 mmoL/L), potassium 2.6 mmoL/L (3.6–5.1 mmoL/L), chloride 85 mmol/L (98–107 mmol/L), bicarbonate 35 mmoL/L (23–31 mmoL/L), calcium 9.4 mg/dL (8.4–10.3 mg/dL) and magnesium 1.2 mg/dL (1.6–2.6 mg/dL). Urine electrolyte levels were as follows: urine sodium 61 mmoL/L, urine potassium 184 mmol/L, urine calcium 3.7 mg/dL (spot urine calcium/creatinine ratio: 0.054), and urine magnesium 10 mg/dL (fractional excretion of magnesium: 13.7%). Urine diuretic screening test was negative. The clinical and laboratory findings were attributed to acquired Gitelman syndrome, and the patient responded well to the initiation of spironolactone, potassium and magnesium supplements. At the peak of her electrolyte losses, the patient required approximately 200–250 mEq potassium chloride and a total of 300 mg of elemental magnesium in divided doses. Her electrolytes were stabilized with ongoing supplementation and a diet rich in potassium and magnesium.

## Discussion

Gitelman syndrome is an autosomal recessive inherited renal disorder characterized by hypomagnesemia, hypokalemia, hypocalciuria and metabolic alkalosis caused by the mutations in the SLC12A3 or CLCNKB genes. These genes are linked to the thiazide sensitive NCCT located on distal convoluted tubule of kidney. Mutational analysis of genes associated with Gitelman syndrome (SLC12A3, CLCNKB) should be performed and genetic testing should be negative, to classify the clinical picture as acquired. However, we did not had access to genetic testing and thus, was not performed. Moreover, congenital Gitelman syndrome usually presents in adolescents or early adulthood and thus was unlikely in our patient given her age.

Treatment options include lifelong administration of drugs that block distal tubule sodium-potassium exchange (e.g., Spironolactone) and supplementation of potassium and magnesium. There is some evidence based on case reports showing that steroids can be beneficial as well [4]. However, our patient was stabilized with aggressive potassium and magnesium repletion. Steroids were not tried given she had active dysphagia from CREST syndrome as part of her scleroderma, and the concern of worsening esophagitis and its attendant gastro intestinal bleeding risk outweighed any potential benefits from steroids.

Acquired Gitelman syndrome secondary to autoimmune diseases has been sparingly described, mostly in the setting of Sjogren’s syndrome, and in one instance, an overlap of Sjogren’s syndrome and scleroderma [5]. To our knowledge, this is the first description of acquired Gitelman syndrome with isolated systemic sclerosis. Given the potential for malignant cardiac arrhythmias from the electrolyte disturbances seen with Gitelman syndrome [6], recognition of this entity and early treatment is important in avoiding these complications in patients with autoimmune diseases, including systemic sclerosis. It is of utmost importance to include renal tubular disorders in the differential diagnosis of autoimmune diseases with electrolyte derangement and renal involvement.

## Conclusions

Acquired Gitelman syndrome is a rare disorder affecting the NaCl cotransporter in the distal tubule of kidney and should be included in the differential of hypokalemic, hypomagnesemic metabolic alkalosis in the setting of an autoimmune disorder. This is a unique case of a 54-year-old woman with systemic sclerosis presenting with serum and urine electrolyte abnormalities attributed to Gitelman syndrome. Acquired Gitelman syndrome has been reported in association with Sjogren syndrome but has never been reported with systemic sclerosis.
